# Reduction in Nesfatin-1 Levels in the Cerebrospinal Fluid and Increased Nigrostriatal Degeneration Following Ventricular Administration of Anti-nesfatin-1 Antibody in Mice

**DOI:** 10.3389/fnins.2021.621173

**Published:** 2021-01-28

**Authors:** Huanhuan Chen, Xuelian Li, Hui Ma, Wei Zheng, Xiaoli Shen

**Affiliations:** ^1^Department of Epidemiology and Health Statistics, Medical School of Qingdao University, Qingdao, China; ^2^School of Health Sciences, Purdue University, West Lafayette, IN, United States

**Keywords:** nesfatin-1, nigrostriatal system, dopaminergic neuron, mitochondrion, Parkinson’s disease, apoptosis, degeneration

## Abstract

Nesfatin-1 is one of several brain-gut peptides that have a close relationship with the central dopaminergic system. Our previous studies have shown that nesfatin-1 is capable of protecting nigral dopaminergic neurons against 1-methyl-4-phenyl-1,2,3,6-tetrahydropyridine (MPTP)-induced neurotoxicity. A recent study also revealed a reduced blood level of nesfatin-1 in patients with Parkinson’s disease (PD). The current study was designed to investigate whether reduced nesfatin-1 in cerebrospinal fluid (CSF) induces nigrostriatal system degeneration. An intra-cerebroventricular (ICV) injection technique was used to administer anti-nesfatin-1 antibody directly into the lateral ventricle of the brain. Enzyme-linked immunosorbent assay (ELISA) results showed that ICV injection of anti-nesfatin-1 antibody into the lateral ventricle of the brain once daily for 2 weeks caused a significant reduction in nesfatin-1 levels in the CSF (93.1%). Treatment with anti-nesfatin-1 antibody resulted in a substantial loss (23%) of TH-positive (TH+) dopaminergic neurons in the substantia nigra pars compacta (SNpc), as shown by immunofluorescence staining, a depletion in dopamine and its metabolites in the striatum detected by high-performance liquid chromatography (HPLC), and obvious nuclear shrinkage and mitochondrial lesions in dopaminergic neurons in the SNpc detected by transmission electron microscopy (TEM). Furthermore, the results from our Western blot and ELISA experiments demonstrated that anti-nesfatin-1 antibody injection induced an upregulation of caspase-3 activation, increased the expression of *p*-ERK, and elevated brain-derived neurotrophic factor (BDNF) levels in the SNpc. Taken together, these observations suggest that reduced nesfatin-1 in the brain may induce nigrostriatal dopaminergic system degeneration; this effect may be mediated *via* mitochondrial dysfunction-related apoptosis. Our data support a role of nesfatin-1 in maintaining the normal physiological function of the nigrostriatal dopaminergic system.

## Introduction

Parkinson’s disease (PD) is one of the most common neurodegenerative diseases in the world (Dawson and Dawson, 2003; de Lau and Breteler, 2006; Elbaz et al., 2016). Most PD patients display motor symptoms, including tremor, muscle rigidity, akinesia (or slow movement), and postural instability; patients also display non-motor symptoms, such as abnormal digestive tract function, mood disorders, and autonomic disturbances (Klockgether, 2004; Beitz, 2014). The clinical pathology includes the loss of dopaminergic neurons in the substantia nigra pars compacta (SNpc) with an ensuing significant reduction in dopamine levels in the striatum (Dauer and Przedborski, 2003; Sarkar et al., 2016; Balestrino and Schapira, 2020). Extensive data in the literature have linked the development of PD to genetic origins, environmental influences, oxidative stress, protein misfolding, and inflammation, among many other factors (Cacabelos, 2017; Delamarre and Meissner, 2017; Boulos et al., 2019). The etiology of PD, however, is not fully understood (Respondek et al., 2019; Bonam and Muller, 2020; Gilmozzi et al., 2020).

Recently, several brain-gut peptides, such as neurotensin, ghrelin, and glucagon-like peptide-1, were identified to play a significant role in regulating the function of the brain dopaminergic system (St-Gelais et al., 2006; Calsolaro and Edison, 2015; Yu et al., 2016). Nesfatin-1, an 82-amino acid polypeptide that is a product of the NEFA/NUCB2 gene identified in 2006, has been shown to have anorexigenic properties (Oh et al., 2006; Stengel et al., 2010; Pałasz et al., 2012). In the brain, nesfatin-1 is expressed mostly in the paraventricular, arcuate, and supraoptic nuclei of the hypothalamus, the nucleus tractus solitarii, the dorsal nucleus of the vagus nerve, and the pituitary gland (Stengel and Taché, 2011; Li et al., 2014). Nesfatin-1 is relatively stable in the blood within 20 min after injection (Pan et al., 2007). Interestingly, this peptide can freely cross the blood-brain barrier in an unsaturated manner (Pan et al., 2007), allowing the delivery of nesfatin-1 into the brain by peripheral injection for the treatment of brain diseases (Dong et al., 2019).

Early studies on nesfatin-1 were mainly focused on its inhibitory effects on eating, weight, and blood glucose regulation (Atsuchi et al., 2010; Su et al., 2010; Goebel et al., 2011; Stengel et al., 2011). Recent reports have also revealed the impacts of nesfatin-1 on reproduction, sleep, anxiety, epilepsy, and depression (Clynen et al., 2014; Kühne et al., 2018; Friedrich et al., 2019; Kaya et al., 2019; Weibert et al., 2019). Özsavcí et al. (2011) were among the first to report that nesfatin-1 exerts neuroprotection against subarachnoid hemorrhage-induced injury in rats by inhibiting neutrophil infiltration and the subsequent release of inflammatory mediators. Tang et al. (2012) further showed that nesfatin-1 significantly suppresses inflammation and neuronal cell apoptosis after head trauma. Our own data also demonstrate that nesfatin-1 is capable of antagonizing rotenone and 1-methyl-4-phenylpyridinium ion (MPP^+^)-induced neurotoxicity, and its neuroprotective effect appears to be associated with the activation of the C-Raf/extracellular signal-regulated kinase (ERK) signaling cascade, leading to reduced apoptosis caused by mitochondrial dysfunction after exposure to the neurotoxic agents rotenone and MPP^+^ (Tan et al., 2015; Shen et al., 2017). More recently, a clinical study provided evidence that the nesfatin-1 level in the blood of PD patients is significantly lower than that in controls (Emir et al., 2019).

While our data, together with data from other groups, have established a protective function of nesfatin-1 in the central nervous system (CNS), the receptor to which nesfatin-1 molecules bind has not yet been identified (Brailoiu et al., 2007, 2013; Shen et al., 2017). Yosten and Samson (2009) postulated that melanocortin 4 receptor (MC4R) may serve as a candidate nesfatin-1 receptor. Brailoiu et al. (2007) further speculated that the receptor of nesfatin-1 is a G-protein-coupled receptor that acts through Gi and Gs. Studies in our lab using the patch clamp technique have demonstrated that nesfatin-1 can directly decrease the excitability of nigral dopaminergic neurons in rat brain slices (Li et al., 2014), thus suggesting that the nesfatin-1 receptor may be expressed in nigral dopaminergic neurons. These findings prompted us to ask whether MC4R is expressed in dopaminergic neurons in the SNpc and whether delivering SHU 9119, an MC4R inhibitor, directly to the lateral ventricle could block MC4R to affect neuronal function in the SNpc through MC4R. The synthetic peptide SHU 9119 {sequence: Ac-Nle-c[Asp-His-DNal(2′)-Arg-Trp-Lys]-NH2} has been generally used to identify the physiological role of MC4R (Hruby et al., 1995; Yang et al., 2002; Grieco et al., 2007).

Based on our own data and data in the literature, we hypothesized that MC4R, a putative nesfatin-1 receptor, is expressed in dopaminergic neurons in the SNpc. We further hypothesized that the level of nesfatin-1 in the central milieu, especially in the cerebrospinal fluid (CSF), is critical to its protective role in maintaining the normal physiological function of the nigrostriatal system. In the present study, we applied an *in vivo* intra-cerebroventricular (ICV) injection technique to deliver SHU 9119 or nesfatin-1 antibody directly into the lateral ventricle of the brain to observe whether neurodegeneration in the nigrostriatal system occurs as a consequence of MC4R receptor inhibition or decreased CSF nesfatin-1 levels.

## Materials and Methods

### Animals

The study was conducted in compliance with standard animal use practices and was approved by the Animal Ethics Committee of Qingdao University (QDU20180120, Jan-2018). Thirty male C57BL/6 mice were purchased from Beijing Vital River Laboratory Animal Technology Co. (Beijing, China). The mice were maintained in a facility with a 12-h light-dark cycle and were provided with food and water *ad libitum*. At the time of use, the mice were 7 weeks old and weighed 20–25 g.

### Experimental Design and Animal Treatment

**FIGURE 1:**
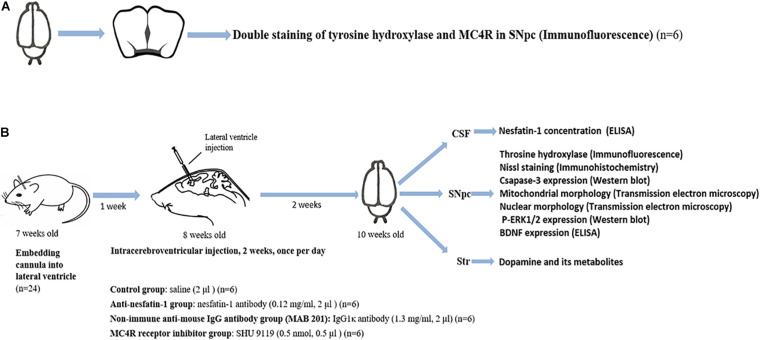
Schematic illustration of the experimental design. **(A)** Double staining of TH and MC4R in the SNpc to demonstrate the presence of MC4R in the SNpc (*n* = 6). **(B)** Anti-nesfatin-1 antibody induces nigrostriatal system degeneration in mice (*n* = 24, six mice per group). The mice received the substances at the doses indicated by ICV injection for 14 days. The CSF was collected for the determination of the nesfatin-1 concentration. The SNpc and striatum were collected for the determination of TH, caspase-3, and *p*-ERK1/2 expression, BDNF concentration, mitochondrial morphology, and nuclear morphology. BDNF, brain-derived neurotrophic factor; CSF, cerebrospinal fluid; ELISA, enzyme-linked immunosorbent assay; MAB 201, non-immune anti-mouse IgG antibody; MC4R, melanocortin 4 receptor; *p*-ERK1/2, phosphorylated ERK1/2; SHU 9119, MC4R receptor inhibitor; SNpc, substantia nigra pars compacta; Str, striatum; TH, tyrosine hydroxylase; ICV, intra-cerebroventricular.

Two sets of experiments were designed to test our hypothesis (Figure 1). Experiment 1 was designed to identify the presence of MC4R on dopaminergic neurons in the SNpc by double staining of tyrosine hydroxylase (TH) and MC4R in brain sections of C57BL/6 mice (*n* = 6) (Figure 1A). Experiment 2 was designed to study whether the lateral ventricle administration of anti-nesfatin-1 antibody would induce nigrostriatal system degeneration in mice (*n* = 24, six mice per group) (Figure 1B). The following is the animal treatment for experiment 2.

To embed the guide cannula into the lateral ventricle, mice were fully anesthetized with chloral hydrate (10%, 10 mL/kg) (Keshi, Chengdu, China) by intraperitoneal (i.p.) injection and placed in a stereotaxic frame (RWD, Shenzhen, China). A longitudinal incision was made in the scalp to expose the surface of the skull. A cranial burr hole (1 mm) was drilled into the skull of the right hemisphere with the following coordinates: 0.3 mm posterior to bregma and 1.0 mm lateral to the midline (Paxinos and Franklin, 2001; Shen et al., 2017, 2020). Next, a stainless steel cannula (RWD, Shenzhen, China) was embedded at 3.2 mm vertical from the skull surface (Young et al., 2012). The cannula was fixed on the skull by a mixture of dental base acrylic resin powder and liquid (Pigeon Dental, Shanghai, China). On the experimental days, the dummy cannula was removed, and an injector (3.2 mm protrusion) was inserted into the guide cannula. ICV injection of drug solutions was performed manually at a rate of 0.5 μL/min (Dore et al., 2017) in freely moving animals through the cannula, which was connected to a 5 μL Hamilton microsyringe (Reno, NV, United States), and the injection cannula was kept *in situ* for an additional 3 min to avoid reflux of the solution along the injector track. The ICV injection technique is well established in this lab and was used in our previous studies (Shen et al., 2017, 2020).

After 1 week of recovery, 24 mice (8 weeks old) were randomly divided into four groups (six mice per group) and administered ICV injections once per day for 14 continuous days of the following: (1) control group: 2 μL saline; (2) non-immune anti-mouse IgG antibody group (MAB 201): 2 μL IgG1κ antibody (1.3 mg/ml) (Millipore, Darmstadt, Germany) (Evans et al., 2019); (3) MC4R receptor inhibitor group: 0.5 μL SHU 9119 (0.5 nmol) (Tocris, Bristol, United Kingdom) (Leckstrom et al., 2009); and (4) anti-nesfatin-1 group: 2 μL nesfatin-1 antibody (0.12 mg/mL) (Phoenix, Burlingame, CA, United States). Twenty-four hours after the last injection, the CSF sample was collected, and then the brain was removed from the skull. The right side of the SNpc was dissected to determine protein levels by Western blot or enzyme-linked immunosorbent assay (ELISA); the striatum was dissected for neurochemical analyses by high-performance liquid chromatography (HPLC). The collected samples were stored at −80°C for future analyses. The left side of the brain was fixed in 4% paraformaldehyde (PFA) for immunofluorescence staining.

### Double Staining of TH and MC4R in the SNpc

This experiment was conducted to identify the presence of MC4R on dopaminergic neurons in the SNpc. Untreated mice (8 weeks old, *n* = 6) were fully anesthetized with chloral hydrate (10%, 10 mL/kg, i.p.) (Keshi, Chengdu, China). Brains of C57BL/6 mice were removed from the skull, fixed in 4% PFA for 72 h at 4°C, incubated in 0.1 mmol/L phosphate buffer (pH 7.4) containing 25% sucrose at 4°C for 2–3 days, and then stored at −80°C. The frozen brain tissues were cut into 20-μm-thick sections. Double immunofluorescence staining is routinely performed in our laboratory (Zhang et al., 2014; Shen et al., 2017). The free-floating brain sections were incubated with 0.3% Triton X-100 diluted in PBS for 2 h at room temperature for permeabilization and blocked in normal goat serum for 1 h at room temperature. The sections were then double-immunostained at 4°C with primary antibodies against TH (1:2,000) (Millipore, Darmstadt, Germany) and MC4R (1:50) (Alomone Labs, Jerusalem, Israel) for 24 h followed by incubation with goat anti-rabbit Alexa 488-conjugated secondary antibody (1:800) (Abcam, Cambridge, United Kingdom) and goat anti-mouse Texas Red secondary antibody (1:800) (Birmingham, AL, United States) at room temperature for 2 h. The brain tissues were mounted on objective slides using ProLong Gold Anti-fade Mountant (Cell Signaling, Boston, MA, United States) to avoid fluorescence bleaching. The negative control was established by using only the secondary antibody to show non-specific background staining. Images were obtained by immunofluorescence microscopy (Observer A1, Zeiss, Germany) at a magnification of 400×.

### CSF Collection

Cerebrospinal fluid samples were collected using a 28G butterfly needle connected to a 1-mL syringe. After being anesthetized with chloral hydrate (10%, 10 mL/kg, i.p.) (Keshi, Chengdu, China), the surface of the mouse brain was held vertically on the work surface, and the needle was inserted vertically (relative to the work surface) between the protuberance and the spine of the atlas. After a puncturing sensation was felt, the CSF was slowly collected (Gu et al., 2012).

### Quantification of Nesfatin-1 in the CSF by ELISA

Cerebrospinal fluid samples from six mice per group were collected for ELISA. CSF samples (10 μL) were diluted 1:20 with artificial CSF (a buffer containing 103 mmol/L NaCl, 4.7 mmol/L KCl, 1.2 mmol/L KH2PO4, 1.2 mmol/L MgSO4, 25 mmol/L NaHCO3, 10 mmol/L glucose, 1 mmol/L sodium pyruvate, and 2.5 mmol/L CaCl2, pH 7.4) (Shen et al., 2020). Concentrations of nesfatin-1 in the CSF were determined by a mouse nesfatin-1 ELISA kit from JianglaiBIO (Beijing, China) according to the manufacturer’s instructions (Zhang et al., 2018).

### Immunofluorescence Staining of TH in the SNpc

Tyrosine hydroxylase is a rate-limiting enzyme in the synthesis process of dopamine and norepinephrine (Moore and Bloom, 1979). It is abundantly expressed in dopaminergic neurons (Raisman-Vozari et al., 1991; Blanchard et al., 1993). The protocol for immunofluorescence staining of TH is routinely used in our laboratory (Jiang et al., 2008; Shen et al., 2017). Brains were fixed in 4% PFA for 72 h at 4°C then incubated in 0.1 mol/L phosphate buffer (pH 7.5) containing 25% sucrose at 4°C for 2–3 days. The frozen brain tissues were cut into 20-μm-thick sections. The brain tissue sections were used for immunofluorescence staining of TH in the SNpc. The free-floating sections were first incubated with 0.1% Triton X-100 and goat serum (Gibco-BRL, Grand Island, NY, United States) in phosphate-buffered saline (PBS) for 2 h and then incubated overnight at 4°C with the TH primary antibody (1:1,000) (Millipore, Burlington, MA, United States) in PBS containing 0.1% Triton X-100 (St. Louis, MO, United States). The sections used for staining TH in the SNpc were incubated with Alexa Fluor 555-conjugated donkey anti-rabbit secondary antibody (Abcam, Cambridge, United Kingdom), and images were obtained by immunofluorescence microscopy (Observer A1, Zeiss, Germany) at magnifications of 100× and 400×.

### Nissl Staining in the SNpc

Brain sections (20 μm) were stained with Nissl staining reagent (Beyotime, Shanghai, China) for 20–30 min and then rinsed with double-distilled water for 5 min, 70% ethanol solution for 5 s, and 95% ethanol solution for 5 s. Then, the brain sections were dehydrated in anhydrous ethanol, cleared with xylene solution (Sinopharm, Shanghai, China), and mounted with neutral gum (Yiyang, Shanghai, China) (Jyothi et al., 2015; Fathalla et al., 2017).

### Stereological Analysis

Total numbers of TH-positive and Nissl-positive neurons were estimated bilaterally from every 4th section through the extent of the SNpc of each brain. Stereology was performed at 400× using an Axioplan 2 imaging microscope (Zeiss, Göttingen, Germany) fitted with a DEI-750 CE video camera (Optronics, CA, United States) and a LEP MAC5000 motorized stage controller (Ludl Electronic Products, NY, United States). The software package used was Stereo Investigator (MBF Bioscience, VT, United States). The coefficient of error for the individual counts was 0.01. A counting frame of 50 μm × 50 μm and a height of 10 μm was chosen. Only the neurons within the counting frame were counted. At least 50 markers were counted within 50 framing sites for each mouse. The neuron count multiplied by four was used as the final number of TH+ cells. Data are expressed as TH(+)-dopaminergic neurons/SNpc (Healy-Stoffel et al., 2012; Park et al., 2012; Nam et al., 2015; Shen et al., 2017; Peng et al., 2018).

### Quantification of Dopamine and Its Metabolite Levels by HPLC

The HPLC protocol has been well established and is routinely used in our laboratory (Jiang et al., 2008; Shen et al., 2017). Samples of the striatum were weighed and then homogenized in 120 μL of solution A (0.4 M perchloric acid). After initial centrifugation (12,000 rpm for 20 min at 4°C) (Eppendorf 5810R, Germany), 80 μL of the supernatant was transferred into Eppendorf tubes, and 40 μL of solution B [containing 20 mM citromalic acid potassium, 300 mM dipotassium phosphate, 2 mM ethylenediamine tetraacetic acid (EDTA)⋅2Na] was added. After additional centrifugation (12,000 rpm for 20 min at 4°C), 100 μL of the supernatant was assayed for dopamine (DA) and its metabolites homovanillic acid (HVA) and dihydroxyphenylacetic acid (DOPAC) by HPLC. Separation was achieved on an Agilent C18 reverse-phase column (4.6 mm × 150 mm × 5 μm) (Agilent, CA, United States). The mobile phase consisted of 20 mM citromalic acid, 50 mM sodium caproate, 0.134 mM EDTA⋅2Na, 3.75 mM sodium octane sulfonic acid, and 1 mM di-sec-butylamine in 5% (v/v) methanol; the flow rate was 1 mL/min. A 2,465 electrochemical detector (Waters, United States) was operated in screen mode. The results are expressed as ng/mg wet weight of brain tissue.

### Western Blot

Samples of the SNpc were lysed in RIPA lysis buffer containing protease inhibitor and phosphatase inhibitor cocktail (Beyotime, Shanghai, China). The protein concentration was determined using BCA kits (Beyotime, Shanghai, China) (Zhang et al., 2020). For Western blotting, the samples were boiled in 5 × loading buffer (Applygen, Beijing, China), electrophoresed on a 12% Tris-glycine gel by sodium dodecyl sulfate-polyacrylamide gel electrophoresis (SDS-PAGE) and then transferred to polyvinylidene fluoride membranes with a pore size of 0.45 μm (Merck Millipore, MA, United States) (Schägger, 2006). After blocking with 7% non-fat milk at room temperature for 2 h, the membranes were incubated for 24 h with primary antibody overnight at 4°C and then with secondary antibodies coupled to horseradish peroxidase for 2 h. The following primary antibodies purchased from Cell Signaling Technology (Boston, MA, United States) were used for Western blot analysis: phospho-ERK1/2 (1:1,000), caspase-3 (1:1,000), and glyceraldehyde-3-phosphate dehydrogenase (GAPDH) (1:1,000). The following secondary antibodies were used for Western blot analysis: anti-rabbit IgG-HRP (1:10,000) and anti-mouse IgG-HRP (1:10,000) (Santa Cruz Biotechnology, Dallas, TX, United States). Cross-reactivity was visualized using ECL Western blot detection reagents (Millipore, Burlington, MA, United States), analyzed by scanning densitometry using UVP VisionWorks^TM^ LS Software (UVP, Cambridge, United Kingdom) and quantified with ImageJ Software (Zhang et al., 2020).

### Morphological Examination of Mitochondria and Nuclei by Transmission Electron Microscopy

The subcellular microstructure was examined by using a JEOL JEM-1400 transmission electron microscope (JEOL, Tokyo, Japan) at the Electron Microscopy Core Facility at Qingdao University. SNpc samples were cut into 2 mm^3^ pieces, fixed with 2.5% glutaraldehyde for more than 4 h, postfixed with 2% osmium tetroxide in Sorensen’s buffer for 1 h, dehydrated in an ascending ethanol series (30, 50, 70, 80, 90, 95, and 100%), and embedded in Epon/Araldite resin. Thin sections (70 nm) were cut using a Leica EM UC7 Ultramicrotome (Leica Microsystems, Wetzlar, Germany) and placed on 100 mesh copper grids (Electron Microscopy Sciences, Hatfield, PA, United States). Sample grids were poststained with premixed solutions of uranyl acetate and lead citrate (Ultrostain I and II, respectively, Leica Microsystems, Wetzlar, Germany) and examined at 80 kV using a JEOL JEM-1400 transmission electron microscope (JEOL, Tokyo, Japan) (Eustaquio et al., 2018).

For animals in each group (*n* = 6 per group), at least three samples per animal were prepared. Images were captured of randomly selected neurons (approximately 10 neurons per animal) from all the animals in each group. The images were recorded using a TVIPS F416 4k × 4k CCD camera running EM-MENU 4.0 acquisition software (Tietz Video and Image Processing Systems, Gauting, Germany). Mitochondrial length (along the long axis) and the number of perinuclear mitochondria were counted and summarized from 10 images for each animal. The nuclear morphology of dopaminergic neurons was also observed and described (Eustaquio et al., 2018).

### Quantification of Brain-Derived Neurotrophic Factor in the SNpc by ELISA

Samples of SNpc were lysed in RIPA lysis buffer containing protease inhibitor and phosphatase inhibitor cocktail (Beyotime, Shanghai, China). The protein concentration was determined using BCA kits (Beyotime, Shanghai, China). The samples were then diluted 1:10 with sample dilution buffer (1% BSA with 0.05% Tween-20). The brain-derived neurotrophic factor (BDNF) concentration was determined by the Total BDNF Quantikine ELISA Kit from R&D Systems (MN, United States) according to the manufacturer’s instructions (Polacchini et al., 2015).

### Statistical Analysis

SPSS 20.0 (SPSS Inc., Chicago, IL, United States) was used to analyze the data. All data are shown as the mean ± SD. Differences between the means of two groups were compared using the unpaired-samples *t*-test. One-way analysis of variance (ANOVA) followed by the Student–Newman–Keuls test was used to compare differences between means in more than two groups. *p* < 0.05 was considered statistically significant.

## Results

### Expression of MC4R in Dopaminergic Neurons in the SNpc

**FIGURE 2:**
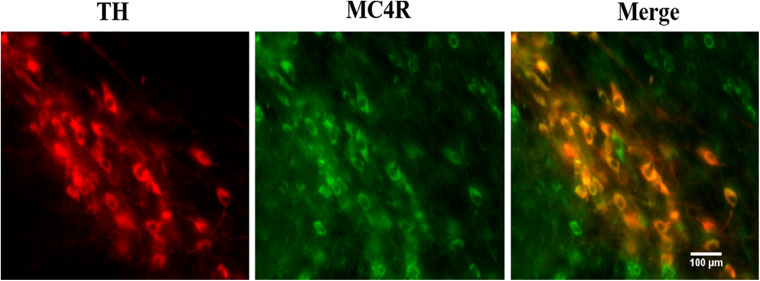
Double staining of TH and MC4R in the mouse SNpc (*n* = 6). The images were obtained by immunofluorescence microscopy. TH, tyrosine hydroxylase; MC4R, melanocortin 4 receptor; SNpc, substantia nigra pars compacta.

Tyrosine hydroxylase is typically expressed in dopaminergic neurons (Pickrell et al., 2015; Shehadeh et al., 2019). A double staining technique was used to detect the expression of MC4R in dopaminergic neurons in the SNpc. The images in Figure 2 illustrate the robust signal of the MC4R staining in the SNpc. The MC4R signals were colocalized with TH signals, indicating the presence of MC4R in dopaminergic neurons in the SNpc.

### Decreased Nesfatin-1 Levels in CSF Following Daily ICV Injection of Anti-nesfatin-1 Antibody

Following the various ICV treatments, we used ELISA to quantify the concentration of nesfatin-1 in the CSF. The data presented in Table 1 show that daily ICV injection of saline, non-immune anti-mouse IgG antibody, MC4R receptor inhibitor (SHU 9119), and anti-nesfatin-1 antibody for 14 days resulted in nesfatin-1 concentrations in the CSF of 0.29 ± 0.083, 0.33 ± 0.019, 0.31 ± 0.065, and 0.02 ± 0.010 ng/mL, respectively. Treatment with anti-nesfatin-1 antibody significantly decreased the nesfatin-1 level in the CSF compared to the control treatment (by 93.1%), non-immune anti-mouse IgG antibody treatment, and MC4R receptor inhibitor treatment (*p* < 0.05).

**TABLE 1:** Nesfatin-1 concentrations in the CSF with or without direct injection of antibody or inhibitor into the lateral ventricle.

Group	Drug	Nesfatin-1 concentration (ng/ml)
Control	Saline	0.29 ± 0.083
Anti-nesfatin-1 antibody	2 μl nesfatin-1 (0.12 mg/ml)	0.02 ± 0.010*
Non-immune anti-mouse IgG antibody	2 μl IgG1κ antibody (1.3 mg/ml)	0.33 ± 0.019
MC4R receptor inhibitor	0.5 μl SHU 9119 (0.5 nmol)	0.31 ± 0.065

The drugs were administered to mice by ICV injection once daily for 14 days. The data represent the mean ± SD. *n* = 6 per group. *p < 0.05, compared to the controls. MC4R, Melanocortin 4 receptor.

### ICV Injection of Anti-nesfatin-1 Antibody Induced Dopaminergic Neuron Loss in the SNpc

**FIGURE 3:**
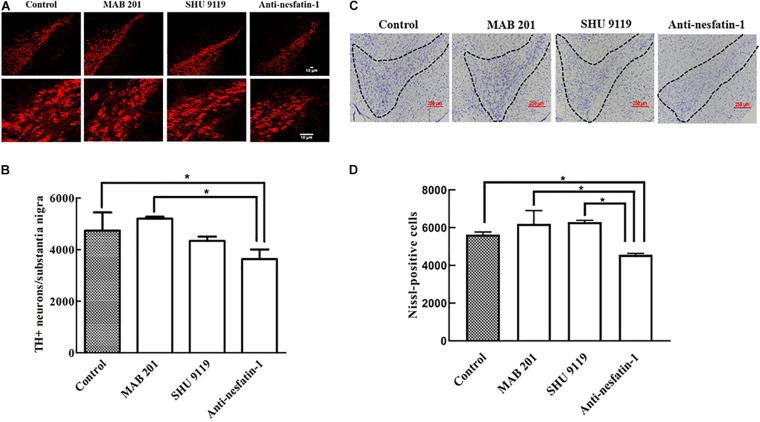
Anti-nesfatin-1 treatment induced nigral dopaminergic neuron degeneration. **(A)** TH(+)-dopaminergic neurons in the SNpc of control mice and mice treated with MAB 201, SHU 9119, or anti-nesfatin-1 antibody are shown. **(B)** A summary of the data showing the numbers of TH(+)-dopaminergic neurons in the different groups (TH, tyrosine hydroxylase). **(C)** Nissl-positive neurons in control mice and mice treated with MAB 201, SHU 9119 or anti-nesfatin-1 antibody are shown. The dotted portion indicates the SNpc. **(D)** A summary of the data showing the numbers of Nissl-positive neurons in the different groups. Each value represents the mean ± SD, *n* = 6; *p < 0.05. MAB 201, non-immune anti-mouse IgG antibody; SHU 9119, MC4R receptor inhibitor; SNpc, substantia nigra pars compacta; TH(+), TH-positive.

After 14 days of daily ICV injection, the numbers of TH-immunopositive (TH+) dopaminergic neurons in the SNpc in the control group, non-immune anti-mouse IgG antibody group, MC4R receptor inhibitor group, and anti-nesfatin-1 group were 4,736 ± 702.73, 5,200 ± 72.32, 4,336 ± 168.57, and 3,632 ± 372.84, respectively. Direct injection of anti-nesfatin-1 antibody into the brain ventricle resulted in a significant loss of TH(+)-dopaminergic neurons in the SNpc (Figures 3A,B). The survival ratio of TH(+)-dopaminergic neurons in the SNpc in the anti-nesfatin-1 group decreased by 23% compared to that in the control group (*p* < 0.05) and by 30% compared to that in the non-immune anti-mouse IgG antibody group (MAB 201 group) (*p* < 0.05). In the MC4R receptor inhibitor (SHU 9119)-treated group, there was no significant reduction in the number of dopaminergic neurons compared with that in the control group.

After 14 days of daily ICV injection, the numbers of Nissl-positive neurons in the SNpc in the control group, non-immune anti-mouse IgG antibody group, MC4R receptor inhibitor group, and anti-nesfatin-1 group were 5,638 ± 132.94, 6,210 ± 698.62, 6,295 ± 94.75, and 4,562 ± 79.37, respectively. Direct injection of anti-nesfatin-1 antibody into the brain ventricle resulted in a significant loss of Nissl-positive neurons in the SNpc (Figures 3C,D). The survival ratio of Nissl-positive neurons in the SNpc in the anti-nesfatin-1 group decreased by 19% compared to that in the control group (*p* < 0.05) and by 26.5% compared to that in the non-immune anti-mouse IgG antibody group (MAB 201 group) (*p* < 0.05).

### ICV Injection of Anti-nesfatin-1 Antibody Induced Depletion of Dopamine and Its Metabolites in the Striatum

**FIGURE 4:**
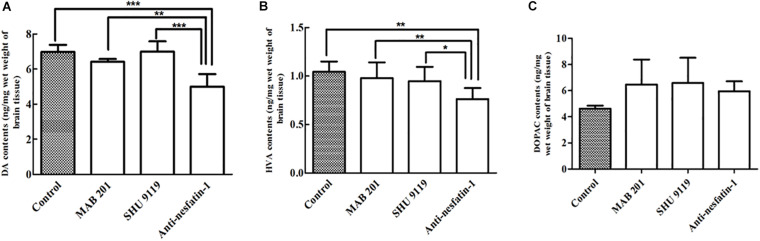
Anti-nesfatin-1 antibody-induced depletion of DA and HVA in the striatum of the right brain (*n* = 6). **(A)** Striatal DA levels in control mice and mice treated with MAB 201, SHU 9119, or anti-nesfatin-1 antibody are shown. **(B)** Striatal HVA levels in the different groups. **(C)** Striatal DOPAC levels in the different groups (DA, dopamine; HVA, homovanillic acid; DOPAC, dihydroxyphenylacetic acid). Each value represents the mean ± SD, *n* = 6; ***p < 0.0001, **p < 0.005, and *p < 0.05. MAB 201, non-immune anti-mouse IgG antibody; SHU 9119, MC4R receptor inhibitor.

After 14 days of daily treatments, the levels of DA and its metabolite HVA were significantly decreased in the striatum in the anti-nesfatin-1 group. Anti-nesfatin-1 antibody treatment resulted in 28, 22, and 29% depletion of DA compared to its levels in the control, non-immune anti-mouse IgG antibody, and MC4R receptor inhibitor groups (*p* < 0.05), respectively (Figure 4A). Anti-nesfatin-1 treatment caused 26, 22, and 19% depletion of HVA, respectively, compared to its level in these groups (*p* < 0.05) (Figure 4B). The DA and HVA levels in mice pretreated with MAB 201 and SHU 9119 did not significantly change compared with those in the control mice (Figures 4A,B). No significant difference was found in DOPAC levels among all the treatment groups (Figure 4C).

### Anti-nesfatin-1 Antibody Treatment Stimulated Caspase-3 Expression in the SNpc

**FIGURE 5:**
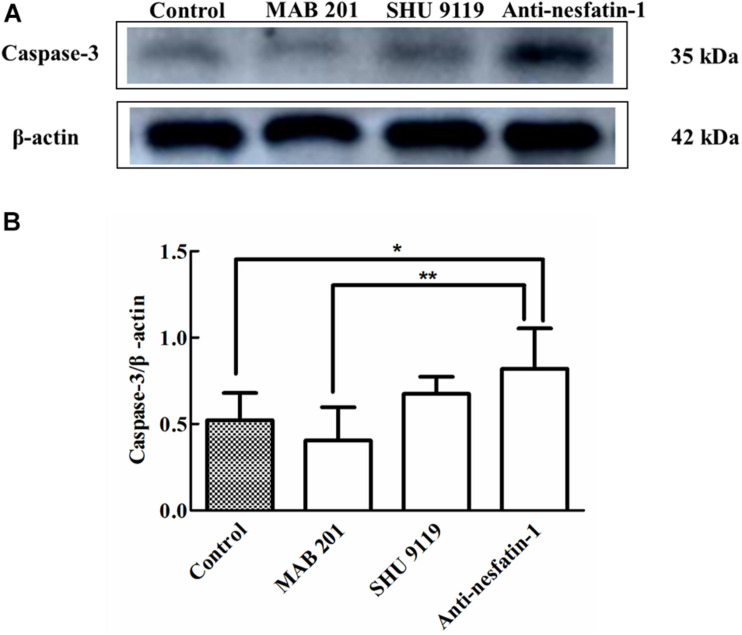
Anti-nesfatin-1 antibody treatment stimulated caspase-3 protein expression in the SNpc (*n* = 6). **(A)** An original image of a Western blot showing the caspase-3 protein levels in control mice and mice treated with MAB 201, SHU 9119, or anti-nesfatin-1 antibody. **(B)** Statistical analysis of caspase-3 protein levels in the different groups. Each value represents the mean ± SD, *n* = 6; **p < 0.005, and *p < 0.05. MAB 201, non-immune anti-mouse IgG antibody; SHU 9119, MC4R receptor inhibitor; SNpc, substantia nigra pars compacta.

Caspase-3 activation is a recognized marker of cell apoptosis (Rogers et al., 2017). The Western blot data in Figure 5A show that ICV injection of the anti-nesfatin-1 antibody increased the expression of caspase-3. After 14 days of daily ICV injection, the ratios of caspase-3/β-actin expression in the SNpc in the control group, non-immune anti-mouse IgG antibody group, MC4R receptor inhibitor group, and anti-nesfatin-1 group were 0.522 ± 0.177, 0.405 ± 0.215, 0.740 ± 0.069, and 0.817 ± 0.260, respectively. The ratios of caspase-3/β-actin expression demonstrated that the anti-nesfatin-1 antibody treatment increased caspase-3 expression by 57% compared to the control treatment (*p* < 0.05) (Figure 5B). Furthermore, the expression of caspase-3 in the anti-nesfatin-1 group was increased by 102% compared to that in the non-immune anti-mouse IgG antibody group (*p* < 0.05) (Figure 5B). These data indicate a statistically significant upregulation of caspase-3 expression, an indicator of induced cell apoptosis, in the SNpc of mice following the reduction of nesfatin-1 in the CSF.

### ICV Injection of Anti-nesfatin-1 Antibody Induced Mitochondrial Lesions and Nuclear Shrinkage in the SNpc

**FIGURE 6:**
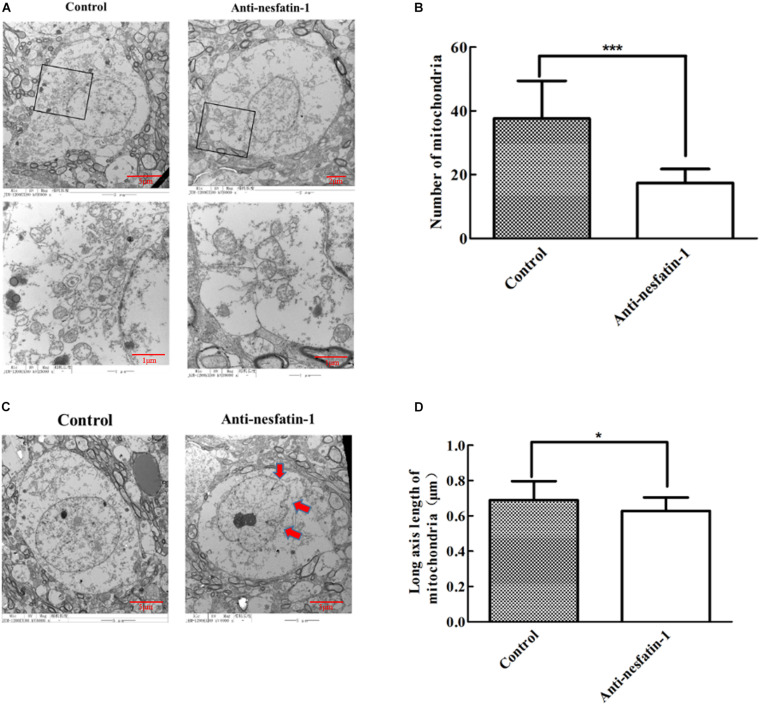
Anti-nesfatin-1 antibody treatment induced mitochondrial depletion and nuclear shrinkage in dopaminergic neurons (*n* = 6). **(A)** Mitochondria in dopaminergic neurons in control and anti-nesfatin-1 antibody-treated mice. **(B)** A summary of the data showing the numbers of mitochondria in the dopaminergic neurons of control and anti-nesfatin-1 antibody-treated mice. **(C)** The anti-nesfatin-1 antibody induced nuclear shrinkage. The red arrows indicate the shrunken nuclei. **(D)** A summary of the data showing the long axis length of mitochondria in the dopaminergic neurons of control and anti-nesfatin-1 antibody-treated mice. Each value represents the mean ± SD, *n* = 6; ***p < 0.0001, and *p < 0.05. MAB 201, non-immune anti-mouse IgG antibody; SHU 9119, MC4R receptor inhibitor.

Altered cell apoptosis could be due to dysfunctional mitochondria in neuronal cells (Nunnari and Suomalainen, 2012; Perier et al., 2012; Franco-Iborra et al., 2016). We used transmission electron microscopy (TEM) to examine mitochondrial morphology. The data in Figure 6A show a marked reduction in mitochondrial numbers in dopaminergic neurons in the SNpc after daily ICV treatment with anti-nesfatin-1 antibody for 14 days. Compared with the control treatment, treatment with anti-nesfatin-1 resulted in a 54% depletion of the number of mitochondria (Figure 6B).

We further examined nuclei in dopaminergic neurons by TEM. The volume of the nucleus in the anti-nesfatin-1 group was significantly reduced; the edges of the nucleus were also visibly folded inward, suggesting that the nuclei of dopaminergic neurons were impaired following anti-nesfatin-1 treatment (Figure 6C). Additionally, there was a 9% decrease in the length of the mitochondrial major axis (Figure 6D) in the anti-nesfatin-1 group compared to that in the control group (*p* < 0.05).

### ICV Administration of Anti-nesfatin-1 Antibody Stimulated ERK1/2 Expression in the SNpc

**FIGURE 7:**
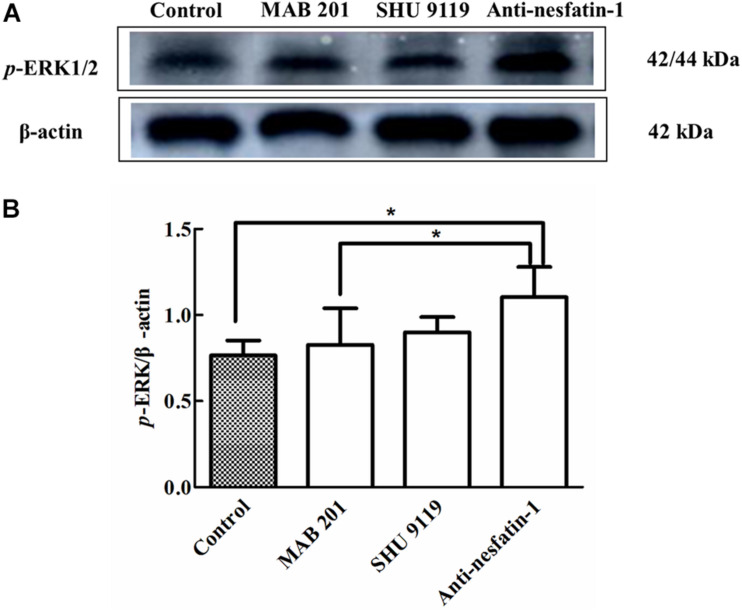
Anti-nesfatin-1 antibody treatment induced an increase in *p*-ERK protein levels (*n* = 6). **(A)** An original image of a Western blot showing the *p*-ERK protein levels in control mice and mice treated with MAB 201, SHU 9119 or anti-nesfatin-1 antibody. **(B)** Statistical analysis of *p*-ERK protein levels in the different groups. Each value represents the mean ± SD, *n* = 6; *p < 0.05. MAB 201, non-immune anti-mouse IgG antibody; SHU 9119, MC4R receptor inhibitor; *p*-ERK, phosphorylated ERK.

Phosphorylation of ERK1/2 is known to activate apoptotic factors within downstream apoptotic pathways, leading to neuronal apoptosis (Jiang et al., 2000). The Western blot data in Figure 7A show that the expression of *p-*ERK1/2 was noticeably increased following anti-nesfatin-1 treatment. After 14 days of treatment, the ratios of *p*-ERK/β-actin in the SNpc in the control group, non-immune anti-mouse IgG antibody group, MC4R receptor inhibitor group, and anti-nesfatin-1 group were 0.766 ± 0.86, 0.827 ± 0.21, 0.900 ± 0.09, and 1.105 ± 0.17, respectively (Figure 7B). The *p*-ERK level in the anti-nesfatin-1 group increased by 44% compared with that in the control group (*p* < 0.05). The *p*-ERK level in the anti-nesfatin-1 group increased by 34% compared to that in the non-immune anti-mouse IgG antibody group. These differences were statistically significant (*p* < 0.05).

### Anti-nesfatin-1 Antibody Treatment Upregulated BDNF Expression in the SNpc

**FIGURE 8:**
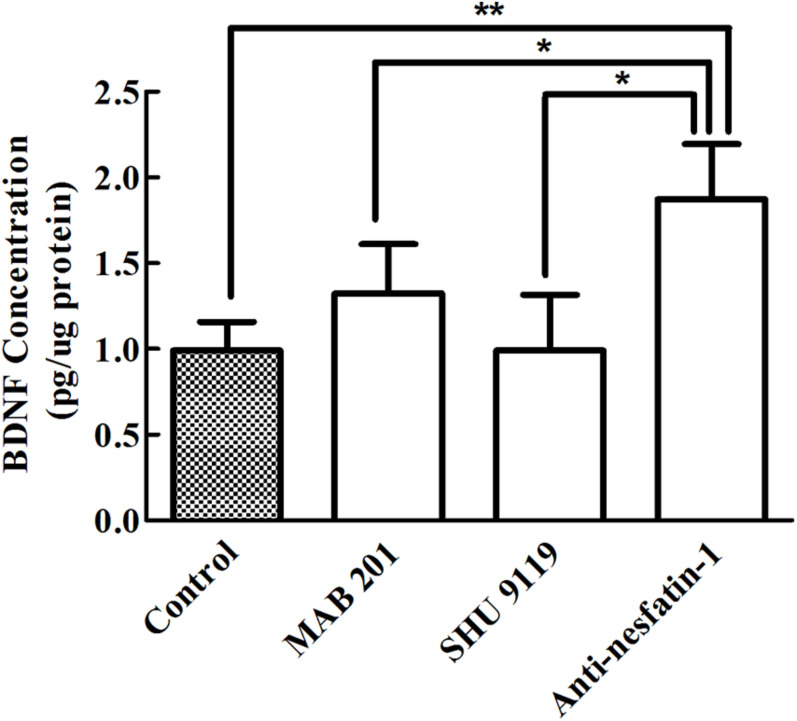
Anti-nesfatin-1 antibody treatment elevated BDNF levels in the SNpc (*n* = 6). Statistical analysis of BDNF protein levels in control mice and mice treated with MAB 201, SHU 9119 or anti-nesfatin-1 antibody. Each value represents the mean ± SD, *n* = 6; **p < 0.005 and *p < 0.05. BDNF, brain-derived neurotrophic factor; MAB 201, non-immune anti-mouse IgG antibody; SHU 9119, MC4R receptor inhibitor; SNpc, substantia nigra pars compacta.

Brain-derived neurotrophic factor is a small-molecule protein structurally related to nerve growth factor that plays an important role in the growth, development, differentiation, maintenance, and regeneration of various types of neurons in the CNS (Allen et al., 2013). ELISA was used to quantify BDNF levels in the SNpc, and as shown in Figure 8, our data demonstrated that after 14 days of treatment, the concentrations of BDNF in the SNpc in the control group, non-immune anti-mouse IgG antibody group, MC4R receptor inhibitor group, and anti-nesfatin-1 group were 0.911 ± 0.17, 1.325 ± 0.29, 0.991 ± 0.33, and 1.873 ± 0.32 pg/μg protein, respectively. A significant increase in the BDNF level in the anti-nesfatin-1 group (by 89%) compared to that in the control group was observed (*p* < 0.05). Similar increases were also observed by comparing the anti-nesfatin-1 group with the non-immune anti-mouse IgG antibody group (41%) and the MC4R receptor inhibitor group (89%); these differences were all statistically significant (*p* < 0.05).

## Discussion

The results presented in this study establish that (1) MC4R, a putative nesfatin-1 receptor, is expressed in dopaminergic neurons in the mouse SNpc; (2) daily ICV injection of nesfatin-1 antibody greatly reduces nesfatin-1 levels in the CSF; (3) a reduced nesfatin-1 level in the CSF is associated with nigrostriatal dopaminergic system degeneration, as evidenced by the reduction in TH(+) neurons, altered DA neurotransmitter levels, and impaired mitochondria and nuclei in the SNpc; and (4) the mechanism underlying DA neuron damage could involve dysfunctional apoptosis. Taking into account the existing evidence in the literature, i.e., the nesfatin-1-mediated rescue of rotenone-induced cell apoptosis in dopaminergic cells (Tan et al., 2015) and MPTP-induced dopaminergic neuron loss in the SNpc (Shen et al., 2017), as well as the reduced nesfatin-1 blood levels in PD patients (Emir et al., 2019), we postulate that the brain peptide nesfatin-1 plays a critical role in maintaining the normal function of the nigrostriatal dopaminergic system.

The main pathological characteristic of PD is the selective loss of TH(+) -dopaminergic neurons in the SNpc, which results in a decrease in the number of nerve fibers projecting from the substantia nigra to the striatum and a subsequent reduction in the release of dopamine from the striatum (Daubner et al., 2011; Alieva et al., 2018). The current study demonstrates that diminished nesfatin-1 in the CSF seems likely to be responsible for dopaminergic neuron degeneration. This statement is supported by the following observations. First, reducing the level of nesfatin-1 in the CSF by administrating anti-nesfatin-1 antibody into the lateral ventricle greatly reduced the numbers of TH(+)-dopaminergic neurons in the SNpc, as shown by our immunofluorescence study. Second, DA and its metabolism in the striatum were significantly reduced after ICV injection of anti-nesfatin-1 antibody, as shown by our HPLC measurements. Furthermore, subcellular structures such as mitochondria and nuclei in dopaminergic neurons were severely damaged in animals treated with anti-nesfatin-1 antibody. Thus, these data support a critical role of nesfatin-1 in the CNS in protecting nigral dopaminergic neurons from degeneration.

To understand the mechanisms by which nesfatin-1 exerts its neuroprotective effects, our first step was to examine whether the diminished CSF nesfatin-1 level induced apoptosis in nigral dopaminergic neurons, since cell apoptosis has been repeatedly shown in the literature to be associated with nigral dopaminergic neuron degeneration in postmortem brain tissues from PD patients (Mochizuki et al., 1996; Anglade et al., 1997). The results from the present study clearly showed that the reduction in nesfatin-1 in the CSF resulting from ICV injection of anti-nesfatin-1 antibody increased the expression of caspase-3 in the SNpc, which suggests that apoptosis was induced in nigral dopaminergic neurons in the SNpc.

Our next step was to evaluate the structural integrity of mitochondria and the nucleus because evidence in the literature has established that damaged mitochondria may lose their ability to produce energy to support normal cell functionality, leading to neuronal apoptosis, which is involved in dopaminergic neuron degeneration (Ryan et al., 2015; Bose and Beal, 2016; Ganguly et al., 2017; Golpich et al., 2017; Larsen et al., 2018; Surmeier, 2018; Grünewald et al., 2019). Data from the PD brain also show that mitochondrial dysfunction can increase the production of reactive oxygen species (ROS) by reducing the supply of adenosine triphosphate and blocking energy production (Iversen and Iversen, 2007; Ryan et al., 2015; Bose and Beal, 2016). The ROS produced in mitochondria can further lead to the opening of mitochondrial permeability transition pores and the hyperpolarization of the mitochondrial membrane (Zorov et al., 2014). In turn, the damaged mitochondrial membrane allows the leakage of cytochrome C into the cytoplasm, which activates caspase-3 and caspase-3 dependent apoptosis (Przedborski and Vila, 2001; Schapira and Jenner, 2011; Subramaniam and Chesselet, 2013). Our TEM data indicated a substantial decline in mitochondrial numbers in dopaminergic neurons and a significantly shortened length of the long axis of mitochondria after the levels of nesfatin-1 in the CSF were significantly reduced. Moreover, the nuclei in the dopamine neurons were also markedly shrunken. Combining the observations of caspase-3 activation, mitochondrial dysfunction, and nuclear shrinkage, we propose that decreased nesfatin-1 in the CNS may cause mitochondrial lesions in dopaminergic neurons, which may subsequently activate the apoptosis cascade and ultimately lead to the degeneration of dopaminergic neurons.

Studies in the literature have shown that ERK is involved in neuronal cell survival (Fei et al., 2015; Jeong et al., 2017; Shi et al., 2017); in particular, the state of ERK1/2 activation may determine whether the kinase promotes cell death or promotes cell survival (Stanciu et al., 2000; Chang and Karin, 2001; Stanciu and DeFranco, 2002; Chu et al., 2004). Yin et al. (2008) demonstrated that chronic manganese exposure in rats results in a significant activation of astrocytic caspase-3 and *p*-ERK that mediates the apoptosis of astrocytes. Our Western blot data revealed a significant activation of *p*-ERK in the SNpc following injection of anti-nesfatin-1 antibody into the CSF. Thus, it seems likely that diminished nesfatin-1 levels in the CNS may weaken the ability of cells to protect mitochondria in dopamine neurons, which may cause the overexpression of *p*-ERK, leading to the apoptosis of dopamine neurons.

Brain-derived neurotrophic factor, a known neurotrophic factor, exerts neuroprotective effects, including anti-apoptotic, and antioxidative effects as well as suppression of autophagy (Wu et al., 2016, 2017; Chen et al., 2017). A significant increase in BDNF levels in the SNpc following the reduction of nesfatin-1 was evident in the current research. Since anti-nesfatin-1 antibody treatment greatly reduced the number of dopaminergic neurons (by 23%), it is possible that the increased BDNF may reflect a compensatory response that offsets the loss of dopaminergic neurons (Hassani et al., 2019).

Finally, does nesfatin-1 exert its neuroprotective effect by acting on its putative receptor, i.e., MC4R? Our previous study showed that nesfatin-1 postsynaptically inhibits the electrical activity of nigral dopaminergic neurons, suggesting that nesfatin-1 receptors are expressed in dopaminergic neurons (Li et al., 2014). A number of candidate nesfatin-1 receptors have been proposed, including MC4R, corticotropin-releasing factor type 2 receptor, and natriuretic peptide receptor A (Yosten and Samson, 2009; Angelone et al., 2013; Ying et al., 2015). Initially, we hypothesized that the effect of nesfatin-1 on dopaminergic neurons may be mediated through its binding to MC4R, while the expression of MC4R in the SNpc was indeed confirmed by our current study. Injection of a specific MC4R inhibitor, SHU 9119, into the lateral ventricle neither caused nigral dopaminergic lesions [i.e., TH(+) -dopaminergic neurons, DA levels] nor induced apoptosis (i.e., caspase-3) or other related signaling pathways (i.e., ERK1/2 and BDNF). Thus, it is highly unlikely that MC4R is involved in the function of nesfatin-1 in protecting dopamine neurons in the SNpc.

In summary, our data demonstrate that reducing the nesfatin-1 concentration in the CSF by administering anti-nesfatin-1 antibody into the lateral ventricle can induce nigrostriatal dopaminergic system degeneration *in vivo*. This effect may be mediated by apoptosis triggered by mitochondrial dysfunction. Our study provides new evidence that nesfatin-1 plays a role in maintaining the normal physiological function of the nigrostriatal system. Further investigation with nesfatin-1 knockout mice is needed to demonstrate the effect of nesfatin-1 on the nigrostriatal dopaminergic system.

## Data Availability Statement

The original contributions presented in the study are included in the article/supplementary material, further inquiries can be directed to the corresponding author/s.

## Ethics Statement

The animal study was reviewed and approved by Animal Ethics Committee of Qingdao University.

## Author Contributions

HC, XL, and HM conducted the experiments. XL wrote the manuscript. WZ revised the manuscript. XS conceived the idea and revised the manuscript. All authors have approved the manuscript.

## Conflict of Interest

The authors declare that the research was conducted in the absence of any commercial or financial relationships that could be construed as a potential conflict of interest.

## Abbreviations

AD: Alzheimer’s disease
ANOVA: one-way analysis of variance
BDNF: brain-derived neurotrophic factor
CNS: central nervous system
CSF: cerebrospinal fluid
DA: dopamine
DOPAC: dihydroxyphenylacetic acid
EDTA: ethylene diaminetetraacetic acid
ELISA: enzyme linked immunosorbent assay
ERK: extracellular signal-regulated kinases GAPDH glyceraldehyde-3-phosphate dehydrogenase
HPLC: high-performance liquid chromatography
HVA: homovanillic acid
ICV: intra-cerebroventricular
MC4R: melanocortin 4 receptor
MPP+: 1-methyl-4-phenylpyridillium ion
MPTP, 1-methyl-4-phenyl-1: 2,3,6-tetrahydropyridine
PBS: phosphate-buffered saline
PD: Parkinson’s disease
*p*-ERK 1/2: phosphorylated ERK1/2
PFA: paraformaldehyde
ROS: reactive oxygen species
SDS-PAGE: sodium dodecyl sulfate-polyacrylamide gel electrophoresis
SNpc: substantia nigra pars compacta
Str: striatum
TEM: transmission electron microscopy
TH: tyrosine hydroxylase
TH(+): TH-immunoreactivity positive
i.p.: intraperitoneal injection.
